# Spectrally Partitioned All-Optical Memristors for Highly Linear Neuromorphic Vision Systems

**DOI:** 10.1007/s40820-026-02331-4

**Published:** 2026-08-31

**Authors:** Junchao Zhang, Bai Sun, Songling Wang, Guangdong Zhou, Linfeng Sun, Zelin Cao, Kaikai Gao, Xiaojun Li, Xiaoliang Chen, Jinyou Shao

**Affiliations:** 1 Frontier Institute of Science and Technology, and Interdisciplinary Research Center of Frontier Science and Technology, Xi’an Jiaotong University, Xi’an, Shaanxi 710049 People’s Republic of China; 2 Micro-and Nano-Technology Research Center, State Key Laboratory for Manufacturing Systems Engineering, Xi’an Jiaotong University, Xi’an, Shaanxi 710049 People’s Republic of China; 3 State Key Laboratory of Structural Chemistry, Fujian Institute of Research on the Structure of Matter, Chinese Academy of Sciences, Fuzhou, 350002 People’s Republic of China; 4 College of Artificial Intelligence, Brain-Inspired Computing & Intelligent Control of Chongqing Key Lab, Southwest University, Chongqing, 400715 People’s Republic of China; 5 Centre for Quantum Physics, Key Laboratory of Advanced Optoelectronic Quantum Architecture and Measurement (MOE), School of Physics, Beijing Institute of Technology, Beijing, 100081 People’s Republic of China

**Keywords:** All-optical memristor, Neuromorphic vision, Brain-like chip, Hardware neural network, Artificial intelligence

## Abstract

All-optical control of synaptic weights offers a promising route toward low-power and massively parallel neuromorphic hardware. However, existing optoelectronic memristors often require electrical assistance or exhibit nonlinear and asymmetric conductance updates, limiting their energy efficiency and scalability. Herein, we report a spectrally partitioned all-optical memristor based on a PbS/PEDOT:PSS/VO_*x*_ heterostructure, in which near-infrared and visible light are coupled to opposite defect-state filling and depletion pathways. Near-infrared illumination selectively excites PbS quantum dots and drives gradual electron transfer into oxygen-vacancy-related defect states in VO_*x*_ through the PEDOT:PSS-regulated interface, producing continuous conductance potentiation. By contrast, visible illumination activates VO_*x*_ and promotes interfacial hole-assisted recombination, leading to reversible depletion of defect-state electrons and conductance depression. This wavelength-selective carrier-transfer process enables highly linear and symmetric all-optical weight updates, achieving a linearity of 0.9994 and an effective 8-bit conductance resolution. The electrical energy consumption per optically induced synaptic event is calculated to be as low as 0.63 fJ under an ultralow probing bias. By integrating the devices into a 32 × 32 all-optically programmed array, we further demonstrate hardware-based BloodMNIST microscopic blood-cell image classification with an accuracy of 96.5% and strong noise robustness. These results provide a viable strategy for developing high-linearity, low-power, and spectrally programmable neuromorphic hardware.

**Supplementary Information:**

The online version contains supplementary material available at https://doi.org/10.1007/s40820-026-02331-4.

## Highlights

- A spectrally partitioned PbS/PEDOT:PSS/VO_*x*_ all-optical memristor enables wavelength-selective potentiation and depression through regulated interfacial carrier dynamics.
- Highly linear bidirectional weight updates with a linearity of 0.9994, 8-bit conductance resolution, and an electrical energy consumption of 0.63 fJ per event are achieved.
- A 32 × 32 memristor array enables hardware-based BloodMNIST inference with 96.5% accuracy and strong noise robustness.

**Supplementary Information:**

The online version contains supplementary material available at https://doi.org/10.1007/s40820-026-02331-4.

## Graphical abstract

**Figure:**
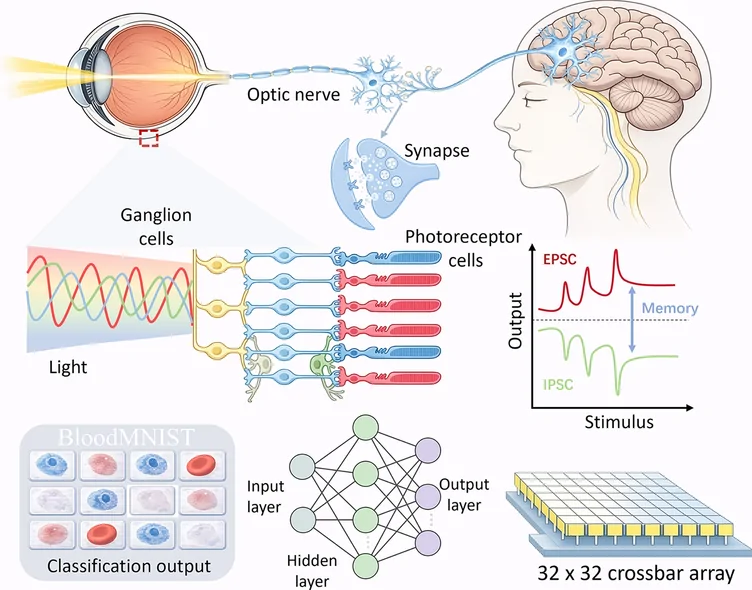

## Introduction

Neuromorphic computing has emerged as a promising paradigm for overcoming the intrinsic limitations of conventional von Neumann architectures in energy efficiency and parallelism [1–3]. By emulating synaptic plasticity in biological neural systems at the hardware level, memristors provide a fundamental device platform for efficient and massively parallel brain-inspired computing systems [4–8]. Meanwhile, the increasing demand for intelligent visual perception has accelerated the development of in-sensor computing architectures, in which sensing, memory, and computation are tightly integrated within the same hardware unit [9–12]. In this context, optoelectronic memristors that directly respond to optical stimuli and modulate synaptic weights have attracted considerable interest as promising building blocks for neuromorphic vision systems [13–18].

Compared with electrically controlled memristors, optoelectronic memristors introduce light as an additional degree of freedom, enabling richer device functionalities and potential system-level simplification [19–22]. However, many reported optoelectronic memristors still rely on cooperative electrical and optical stimulation for conductance modulation [23–25]. Such hybrid operation increases energy consumption and peripheral-circuit complexity, while limiting scalability toward large-area and massively parallel arrays [26, 27]. Although all-optical memristors can reduce electrical intervention during weight programming, their conductance modulation often suffers from pronounced nonlinearity, limited reversibility, or rapid saturation, making it difficult to meet the requirements of high-precision neural networks for linear, stable, and symmetric weight updates [28–31]. Moreover, most existing devices operate within a single spectral-response window, which restricts wavelength-programmable bidirectional modulation, such as potentiation and depression within the same device [32–35]. These limitations remain a major obstacle to scalable, energy-efficient, and spectrally programmable neuromorphic vision hardware.

From a materials perspective, coupling photosensitive semiconductors with defect-state engineering provides an effective route for optically controlled memristive modulation [36–39]. In particular, transition-metal oxides containing abundant oxygen vacancies are attractive because their continuously distributed defect states can regulate carrier transport and enable tunable conductivity. Nevertheless, without rational interfacial regulation, photogenerated carriers are prone to uncontrolled injection or recombination, resulting in abrupt conductance changes, poor update linearity, and degraded cycling stability [40–42]. These problems become more critical when devices are extended from individual cells to large-area fabrication and array-level integration [43–45]. Therefore, a key challenge for all-optical memristors is to achieve reversible, gradual, and wavelength-selective carrier modulation while preserving material scalability and process compatibility.

Motivated by these challenges, we develop a spectrally partitioned, all-optical programmable memristor based on a PbS/PEDOT:PSS/VO_*x*_ heterostructure. Here, “spectrally partitioned” refers not simply to multiband photoresponse, but to the wavelength-selective activation of different carrier-dynamics pathways within one heterostructure. By integrating a near-infrared-absorbing PbS quantum-dot layer with an oxygen-vacancy-rich VO_*x*_ thin film, the device enables distinct optical responses at different wavelengths. The PEDOT:PSS interlayer, rather than serving as the primary photoabsorber, regulates interfacial carrier transport and suppresses abrupt carrier injection, allowing wavelength-dependent carrier dynamics to be separated and coordinated. As a result, near-infrared illumination mainly drives PbS-mediated electron generation and gradual defect-state filling in VO_*x*_, leading to continuous conductance potentiation, whereas visible-light excitation promotes VO_*x*_-related hole generation, interfacial recombination, and defect-state electron depletion, resulting in reversible conductance suppression. In this way, the two opposite conductance-modulation pathways are effectively partitioned in the wavelength domain.

Benefiting from this heterostructure design, the device exhibits highly linear and symmetric conductance updates, stable multilevel conductance modulation under all-optical programming, and an ultralow electrical energy consumption per optically induced synaptic event under an ultralow probing bias, providing a reliable device platform for high-precision neuromorphic computing. Moreover, the process-compatible heterostructure enables uniform fabrication and integration into a 32 × 32 all-optically programmed memristor array. Using this array, we demonstrate hardware-based BloodMNIST microscopic blood-cell image classification with high recognition accuracy and strong robustness against noise perturbations. Taken together, these results indicate that the proposed spectrally partitioned all-optical memristor offers not only a high-linearity synaptic device platform, but also a viable hardware route toward low-power and spectrally programmable neuromorphic vision systems.

## Experimental Section

### Materials

PbS colloidal quantum dots were synthesized using a conventional hot-injection method. Briefly, PbO (0.36 g), oleic acid (1 mL), and 1-octadecene (15 mL) were loaded into a 50-mL three-neck round-bottom flask and heated to 150 °C under a nitrogen atmosphere to form a clear precursor solution. A sulfur precursor solution containing bis(trimethylsilyl) sulfide in 1-octadecene was then rapidly injected into the flask to initiate the nucleation and growth of PbS quantum dots. After the reaction, the mixture was naturally cooled to room temperature, and the quantum dots were purified through repeated acetone precipitation and centrifugation to remove unreacted precursors and excess ligands. The purified PbS quantum dots were finally dispersed in toluene to obtain a stable colloidal solution with a concentration of 5 mg mL⁻^1^. The PEDOT:PSS solution (Clevios™ P VP AI 4083, Heraeus, Germany) was used as received without further purification.

### Device Fabrication

The PbS/PEDOT:PSS/VO_*x*_ heterostructured all-optical synaptic devices were fabricated through a multilayer thin-film stacking process. For single-device fabrication, Al/Au bilayer bottom electrodes with a total nominal thickness of approximately 50 nm were deposited on cleaned Si/SiO_2_ substrates by thermal evaporation through a patterned shadow mask. A thin Al layer was first deposited as an adhesion layer, followed by the deposition of Au to ensure good electrical conductivity and chemical stability. The VO_*x*_ thin films were deposited by radio-frequency (RF) magnetron sputtering using a high-purity vanadium metal target (≥ 99.99%) under a reactive Ar/O_2_ mixed atmosphere. Prior to deposition, the chamber was evacuated to a base pressure below 5 × 10^–4^ Pa. During sputtering, the Ar/O_2_ flow ratio was maintained at 10:1, the working pressure was fixed at 1 Pa, and the RF power was set to 80 W. Under these conditions, VO_*x*_ films with a thickness of approximately 80 nm were obtained. The as-deposited VO_*x*_ films exhibited an amorphous structure, which is favorable for forming continuously distributed oxygen-vacancy-related defect states. No post-deposition annealing was applied.

The PEDOT:PSS interlayer was deposited using the commercial Clevios™ P VP AI 4083 solution. A two-step spin-coating process was employed: The solution was first spread at 1000 rpm with an acceleration of 200 rpm s⁻^1^ for 5 s, followed by spinning at 5000 rpm with an acceleration of 500 rpm s⁻^1^ for 50 s to form a uniform thin film. After spin coating, the samples were annealed on a hot plate at 100 °C for 10 min to remove residual solvent and stabilize the film morphology. The resulting PEDOT:PSS layer thickness was approximately 15 nm. Owing to the high PSS content, hole-transporting nature, and unfavorable electron-transport energy level of AI 4083, this layer served as an electron-blocking and interfacial carrier-regulation layer in the heterostructure. PbS quantum-dot films were deposited onto the PEDOT:PSS layer by spin coating at 5000 rpm for 30 s. After deposition, the samples were annealed at 60 °C for 10 min to form continuous thin films with a final thickness of approximately 10 nm. The resulting PbS layer served as the primary near-infrared absorption layer in the heterostructure, providing photogenerated charge carriers under optical stimulation. Finally, top Au electrodes with a nominal thickness of approximately 10 nm were deposited by thermal evaporation through a patterned shadow mask aligned orthogonally to the bottom electrodes, defining the active cross-point area and completing the vertical device structure.

### Crossbar Array Fabrication

Based on the single-device fabrication process, a 32 × 32 memristor crossbar array was further constructed using a shadow-mask-assisted deposition process. First, vertical bottom electrode lines were formed on cleaned Si/SiO_2_ substrates by thermal evaporation through a patterned shadow mask. Al/Au bilayer electrodes with a total nominal thickness of approximately 50 nm were used as the bottom electrodes. Subsequently, the VO_*x*_, PEDOT:PSS, and PbS functional layers were sequentially deposited to form a continuous PbS/PEDOT:PSS/VO_*x*_ heterostructure. Finally, horizontal top Au electrode lines with a nominal thickness of approximately 10 nm were deposited by thermal evaporation through another patterned shadow mask placed orthogonally to the bottom electrode lines. Therefore, the crossbar array consisted of orthogonally patterned metal lines rather than a continuous metal film covering the entire active layer. This electrode geometry leaves optically accessible active-layer regions and electrode-edge regions around each cross-point, allowing incident light to interact with the PbS/PEDOT:PSS/VO_*x*_ heterostructure during optical programming. All array devices were fabricated within the same batch. The array yield was evaluated by statistically assessing whether each cross-point device exhibited stable and reversible near-infrared-induced potentiation and visible-light-induced depression behaviors, reflecting the functional yield of the array.

### Materials Characterization

The morphology and microstructure of the PbS/PEDOT:PSS/VO_*x*_ heterostructures were characterized by scanning electron microscopy (SEM), energy-dispersive X-ray spectroscopy (EDS), transmission electron microscopy (TEM), and atomic force microscopy (AFM). SEM and EDS were used to analyze the cross-sectional structure and elemental distribution of the device, while TEM was employed to characterize the morphology and crystallinity of PbS quantum dots. AFM was used to evaluate the surface morphology and roughness of the functional films. The optical absorption spectra of VO_*x*_ and PbS films were measured using a UV–Vis–NIR spectrophotometer to evaluate their wavelength-dependent absorption characteristics. Electron paramagnetic resonance (EPR) measurements were performed to investigate oxygen-vacancy-related defect states in VO_*x*_ under different illumination conditions. Photoluminescence (PL) spectroscopy was used to analyze the interfacial carrier-transfer behavior in the PbS/PEDOT:PSS/VO_*x*_ heterostructure.

Illumination-dependent V 2*p* X-ray photoelectron spectroscopy (XPS) spectra were collected from PbS/PEDOT:PSS/VO_*x*_ heterostructures under dark conditions and after 980 or 525 nm illumination treatments. Three samples fabricated in the same batch were used for comparison. For the light-treated samples, continuous 980 nm illumination with a power density of 10.2 mW cm^−2^ was applied for 60 s, while continuous 525 nm illumination with a power density of 4.6 mW cm^−2^ was applied for 60 s. These illumination treatments were used to induce sufficiently detectable valence-state variations for XPS analysis, while the same wavelengths were used as those in the representative optical programming measurements. To access the buried VO_*x*_ layer, the upper PbS/PEDOT:PSS layers were gently removed by controlled low-energy Ar^+^ sputtering before XPS analysis, and identical sputtering conditions were used for all samples. The XPS spectra were collected using a monochromatic Al Kα X-ray source. The binding energies were calibrated using the C 1*s* peak at 284.8 eV, and the relative V^4+^/V^5+^ percentages were calculated from the integrated areas of the deconvoluted V 2*p* peaks. Accordingly, the XPS analysis was used to compare relative illumination-induced changes in vanadium valence states and oxygen-vacancy-related electronic states, rather than to determine the absolute bulk stoichiometry of VO_*x*_.

### Electrical and Optoelectronic Measurements

Electrical and optoelectronic synaptic characterizations were conducted at room temperature. Unless otherwise specified, the programmed conductance states were electrically read at a bias voltage of 0.1 V to ensure an adequate signal-to-noise ratio and reliable differentiation between adjacent conductance states. This read bias was used only for electrical probing and was not involved in device programming. For the transient low-current photoresponse measurement shown in Fig. 3c (later), an ultralow probing bias of 0.001 V was applied to demonstrate that the optically induced incremental current response could still be detected under a small electrical bias. The corresponding electrical energy consumption per optically induced synaptic event was calculated from the applied probing bias, the photoinduced incremental current response, and the optical pulse width. Postsynaptic current signals were recorded using a Keysight B2901B semiconductor parameter analyzer. Optical stimuli were provided by externally controlled light sources. Near-infrared light with a wavelength of 980 nm was used to induce synaptic potentiation, whereas visible light with a wavelength of 525 nm was employed to induce synaptic depression. The optical-pulse power density and pulse width were controlled within the mW cm^−2^ and millisecond ranges, respectively. Light intensity was calibrated using a power meter, and the spatial uniformity and temporal stability of illumination were maintained throughout the measurements.

### Optical Programming and Weight-Update Protocol

Synaptic weight modulation, represented by device conductance modulation, was achieved exclusively through optical pulses. Successive near-infrared light pulses gradually increased the device conductance, corresponding to long-term potentiation (LTP), whereas successive visible-light pulses gradually decreased the conductance, corresponding to long-term depression (LTD). By precisely controlling the number of optical pulses and illumination parameters, multilevel, reversible, and gradual conductance modulation was realized. The linearity, dynamic range, and stability of the programmed conductance states were statistically evaluated over repeated optical programming cycles to exclude random noise and incidental fluctuations. The reliability of the programmed weight states was evaluated through signal-to-noise ratio (SNR) analysis and repeated-read noise measurements. For the noise-limited resolution analysis of adjacent conductance states, a 3σ criterion was adopted, where the minimum resolvable current difference was defined as three times the combined RMS noise of two neighboring states.

### Neuromorphic Array Measurement and Hardware Platform

The array-level experimental platform consisted of FPGA-based control logic, digital-to-analog converters (DACs), word-line drivers and multiplexers (MUXs), the memristor crossbar array, transimpedance amplifiers (TIAs), and analog-to-digital converters (ADCs). The FPGA coordinated array addressing, optical programming timing, and electrical-readout operations. Spatially programmable optical writing was implemented using a digital micromirror device (DMD). The programming light was patterned by the DMD into predefined binary optical patterns and projected onto the 32 × 32 crossbar array through an imaging lens system. Before optical programming, the projected DMD pattern was spatially calibrated and aligned with the optical microscope image of the array using the electrode-line positions as alignment references. During programming, only the DMD micromirror regions corresponding to the selected cell or selected array region were switched to the ON state, while the surrounding regions were kept in the OFF state, enabling localized optical addressing and minimizing unintended illumination of adjacent cells. After optical programming, the conductance states were electrically read using FPGA-controlled row/column multiplexing and peripheral readout circuits. The readout currents were converted into voltage signals by TIAs and subsequently digitized using 10-bit ADCs before being transferred to the control unit for data processing and neural network inference.

### Neural Network Mapping and Dataset

The BloodMNIST dataset, consisting of microscopic blood-cell images, was used for neural network validation. After preprocessing, the images were fed into a convolutional neural network-inspired architecture for feature extraction and classification. The convolution kernels were implemented using the multilevel conductance states of the memristor array, while the fully connected layer weights were represented using a differential conductance scheme:

$$\Delta G = G^{ + } - G^{ - }$$

The neural network weights were first trained in a software environment and then mapped onto the experimentally accessible conductance states of the memristor array for hardware inference. To evaluate the influence of weight quantization on network performance, classification accuracy was tested under 8-bit, 6-bit, 4-bit, and 2-bit weight resolutions and compared with the floating-point software baseline.

## Results and Discussion

### Device Architecture, Material Characterization, and Array Integration of All-Optical Memristors

**Fig. 1:**
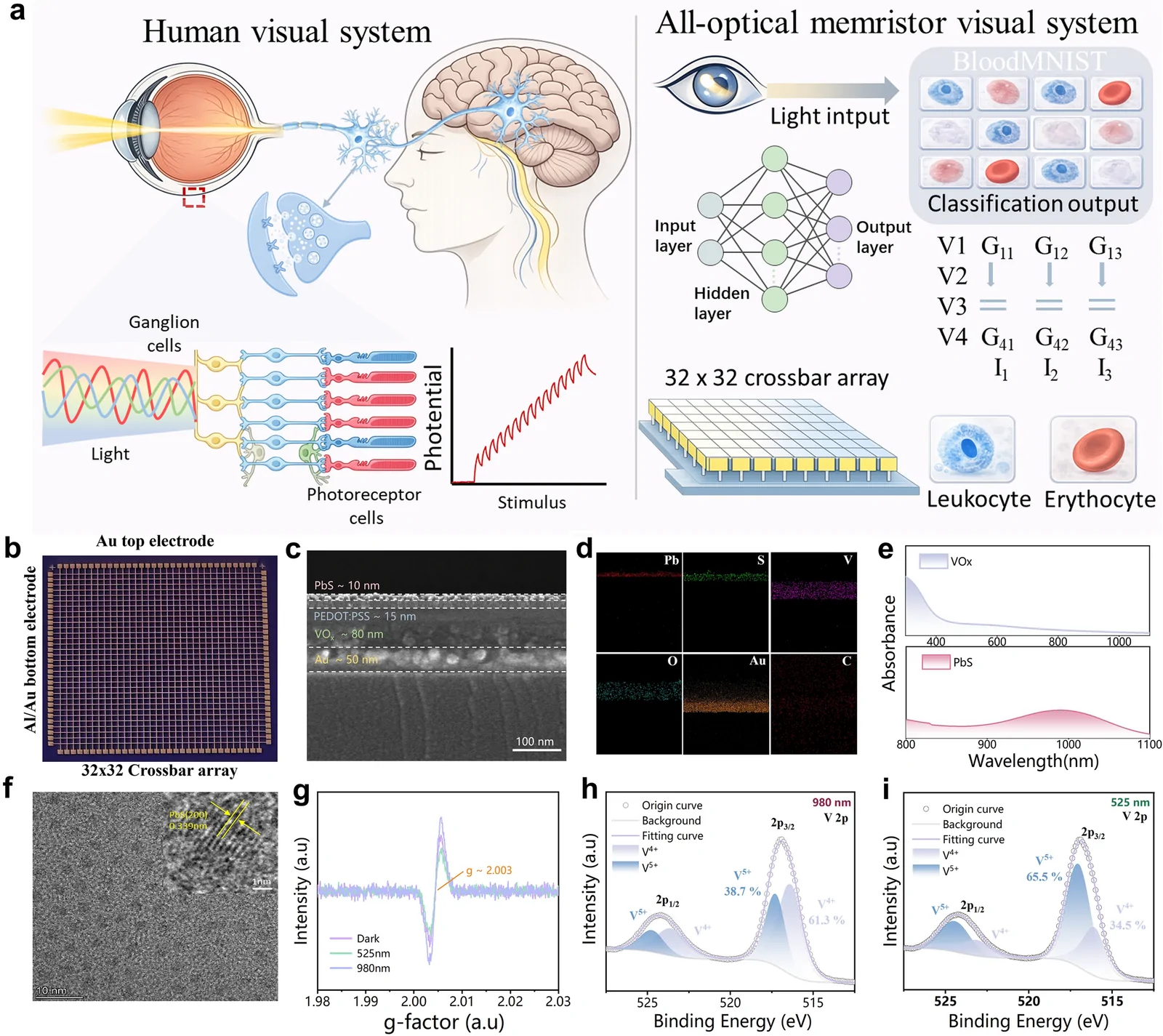
Bioinspired system concept, array implementation, and structural/spectroscopic characterization of the PbS/PEDOT:PSS/VO_*x*_ all-optical memristor. **a** Bioinspired schematic of the optically programmed neuromorphic vision system for BloodMNIST image classification. **b** Optical image of the fabricated 32 × 32 memristor crossbar array. **c** Cross-sectional SEM image of the vertically stacked PbS/PEDOT:PSS/VO_*x*_ heterostructure. **d** Cross-sectional EDS elemental mapping of Pb, S, V, O, Au, and C in a reference heterostructure without the top Au electrode. **e** Optical absorption spectra of VO_*x*_ and PbS films. **f** HRTEM image of PbS quantum dots with lattice fringes corresponding to the PbS (200) plane. **g** EPR spectra under dark, 980, and 525 nm illumination conditions. **h**, **i** High-resolution V 2*p* XPS spectra after 980 and 525 nm illumination, respectively, showing opposite changes in the relative V^4+^/V^5+^ fractions

As illustrated in Fig. 1a, the proposed all-optical memristor visual system is inspired by the human visual pathway. In the biological visual system, external light is received by photoreceptor cells in the retina, converted into neural signals through retinal neurons and ganglion cells, and then transmitted to the brain for visual information processing. Analogously, in the artificial system, incident light serves as the input stimulus for the all-optical memristor array, where optical signals are encoded into tunable conductance states. These conductance states form a hardware weight matrix and participate in neural network computation for BloodMNIST image classification. In this way, the proposed system establishes a bioinspired correspondence between optical perception, synaptic weight modulation, and visual information processing.

To implement this all-optical visual architecture, we constructed a PbS/PEDOT:PSS/VO_*x*_ heterostructured memristor and examined its feasibility for array-level integration. Based on this heterostructure, a 32 × 32 memristor crossbar array was fabricated, as shown in Fig. 1b. The well-ordered crossbar layout and good structural uniformity suggest that the proposed device configuration is suitable for integrated neuromorphic hardware implementation. The cross-sectional SEM image in Fig. 1c further confirms the vertically stacked device architecture, consisting of a PbS quantum-dot absorption layer, a PEDOT:PSS interfacial regulation layer, a VO_*x*_ functional layer, and a bottom-Au electrode. The thicknesses of the PbS, PEDOT:PSS, VO_*x*_, and Au layers are approximately 10, 15, 80, and 50 nm, respectively, consistent with the designed heterostructure.

The elemental distribution of the multilayer stack was further examined by cross-sectional EDS mapping using a reference heterostructure sample prepared without the top Au electrode. As shown in Fig. 1d, the Pb signal is concentrated near the upper region of the heterostructure, corresponding to the PbS quantum-dot layer. By contrast, the S-related distribution is broader because it contains contributions from both PbS and the sulfur-containing PEDOT:PSS interlayer. The V-rich region corresponds to the VO_*x*_ layer, while the Au signal is associated with the conductive layer of the bottom electrode. Together with the EDS line-scan profile in Fig. S1, these results support the sequential PbS/PEDOT:PSS/VO_*x*_/bottom-Au stacking structure.

The optical absorption characteristics of VO_*x*_ and PbS were then investigated to establish the material basis for spectrally partitioned modulation. As shown in Fig. 1e, VO_*x*_ exhibits pronounced absorption in the visible region, whereas PbS shows enhanced absorption in the near-infrared region. This complementary spectral response enables different wavelengths to preferentially activate distinct photoinduced carrier processes within the heterostructure. Specifically, visible-light excitation is mainly associated with the VO_*x*_-related photoresponse, while near-infrared illumination can be effectively absorbed by PbS quantum dots. Such spectral selectivity provides the physical foundation for wavelength-separated optical depression and potentiation within the same memristive device.

The microstructure and crystallinity of the PbS quantum dots were further characterized by TEM. The HRTEM image in Fig. 1f displays clear lattice fringes with an interplanar spacing of approximately 0.339 nm, corresponding to the PbS (200) plane, confirming the crystalline nature of the quantum dots. More detailed TEM characterization is provided in Fig. S2, where the PbS quantum dots exhibit a uniform distribution without obvious aggregation. The corresponding particle-size statistics indicate that the quantum dots are mainly distributed in the range of 4–6 nm with a relatively narrow size distribution, which is beneficial for achieving stable and reproducible near-infrared photoresponses.

The surface morphology of the functional films was evaluated by AFM to assess the interface quality of the heterostructure. As shown in Fig. S3, both the VO_*x*_ and PEDOT:PSS/PbS films exhibit continuous surface morphologies. The pristine VO_*x*_ film shows an RMS roughness of approximately 7.26 nm, whereas the RMS roughness decreases to approximately 3.78 nm after sequential coating with PEDOT:PSS and PbS. This reduced surface roughness indicates that the polymer/QD coating helps form a smoother and more uniform interface, which is favorable for stable interfacial charge regulation and reproducible optoelectronic switching. Besides, to probe the role of oxygen-vacancy-related defect states in optical conductance modulation, EPR measurements were performed under different illumination conditions. As shown in Fig. 1g, the EPR signal centered at g ~ 2.003 can be assigned to unpaired electrons associated with oxygen vacancies in VO_*x*_ [46, 47]. Notably, the signal intensity increases under 980 nm illumination but decreases under 525 nm illumination compared with the dark state. This opposite evolution indicates that near-infrared light and visible light induce distinct changes in the electronic occupation of oxygen-vacancy-related defect states.

High-resolution V 2*p* XPS spectra provide further evidence for the wavelength-dependent modulation of oxygen-vacancy-related electronic states. As shown in Fig. 1h, after 980 nm illumination, the relative fraction of V^4+^ increases to 61.3%, while the V^5+^ component decreases to 38.7%, indicating electron filling of oxygen-vacancy-related defect states in VO_*x*_. In contrast, after 525 nm illumination, the V^5+^ fraction increases to 65.5%, accompanied by a decrease in the V^4+^ fraction to 34.5% (Fig. 1i), suggesting depletion of defect-state electrons. Compared with the pristine VO_*x*_ state shown in Fig. S4, where V^4+^ and V^5+^ account for 52.8% and 47.2%, respectively, these opposite changes under 980 and 525 nm illumination quantitatively support the proposed wavelength-dependent defect-state filling and depletion mechanism. The survey XPS spectrum of the PEDOT:PSS/PbS film and the initial V 2*p* spectrum of pristine VO_*x*_ are provided in Fig. S4, confirming the elemental composition of the functional layers and the coexistence of mixed V^4+^/V^5+^ states in the as-prepared VO_*x*_ film.

Taken together, these structural, optical, and spectroscopic results demonstrate that the PbS/PEDOT:PSS/VO_*x*_ heterostructure provides an effective material platform for spectrally partitioned all-optical conductance modulation. In this architecture, PbS functions as the near-infrared absorption layer, VO_*x*_ provides oxygen-vacancy-related defect states for conductance tuning, and PEDOT:PSS serves as an interfacial carrier-regulation layer. The coordinated roles of these layers enable wavelength-dependent defect-state filling and depletion, thereby laying the foundation for the highly linear bidirectional optical weight modulation and array-level neuromorphic vision functions discussed below.

### Spectrally Partitioned All-Optical Synaptic Plasticity and Carrier-Dynamics Mechanism

**Fig. 2:**
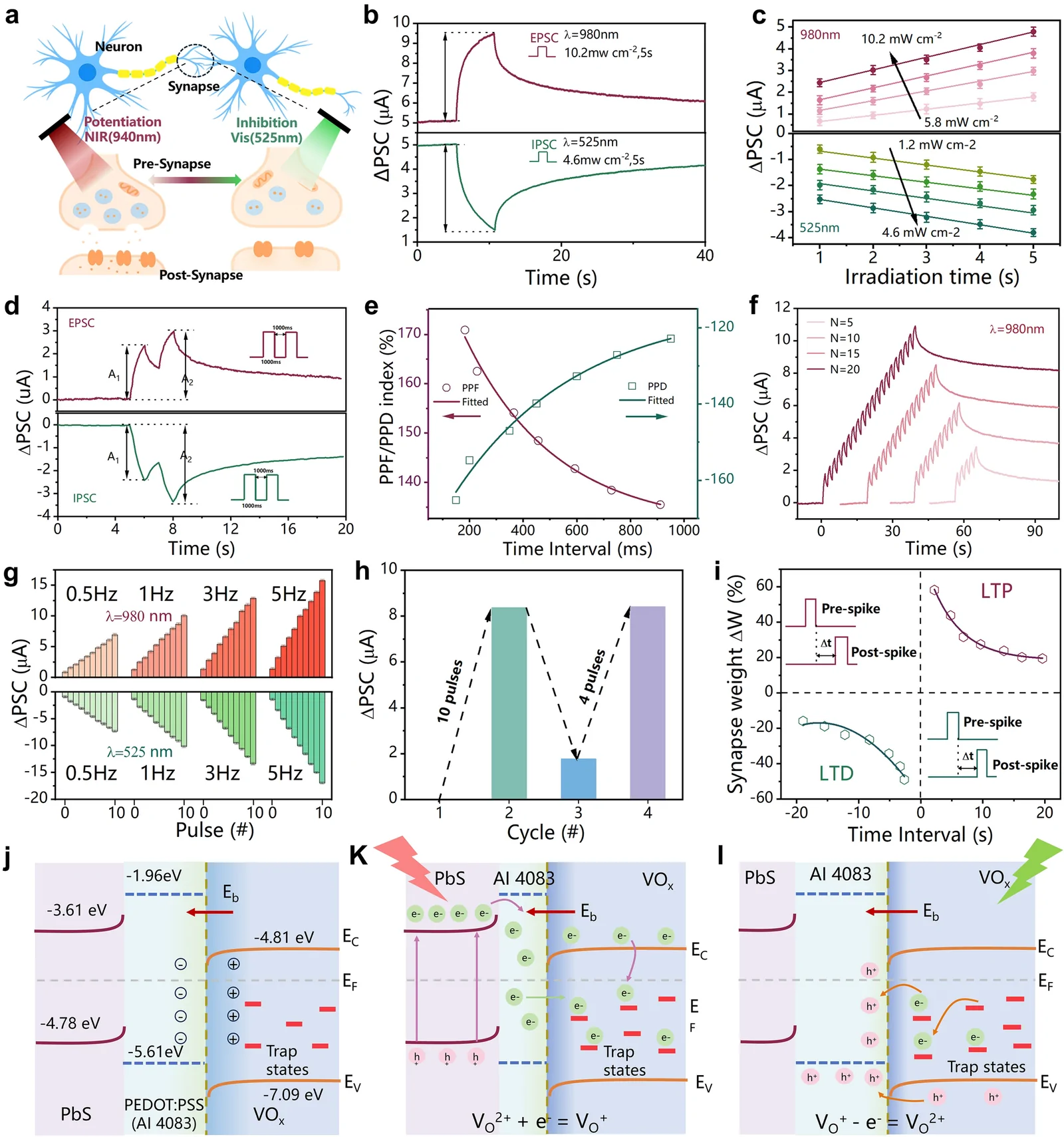
Spectrally controlled all-optical synaptic plasticity of the PbS/PEDOT:PSS/VO_*x*_ device. **a** Schematic illustration of the biological synapse analogy, where near-infrared and visible light induce synaptic potentiation and depression, respectively. **b** EPSC induced by a single 980 nm optical pulse and IPSC induced by a single 525 nm optical pulse. **c** EPSC and IPSC modulation as a function of illumination time under different light intensities. **d** Paired-pulse facilitation (PPF) and paired-pulse depression (PPD) under paired optical pulse stimulation. **e** PPF/PPD index as a function of pulse interval. **f** Cumulative EPSC enhancement under different numbers of 980 nm optical pulses. **g** Frequency-dependent synaptic responses under optical pulse trains with different repetition frequencies. **h** Learning–forgetting–relearning behavior under cyclic optical pulse stimulation. **i** STDP learning window showing LTP and LTD behaviors. **j** Energy-band alignment of the PbS/PEDOT:PSS/VO_*x*_ heterostructure. **k**, **l** Schematic illustration of defect-mediated conductance potentiation under near-infrared illumination and conductance suppression under visible-light illumination, respectively

In biological synapses, excitatory and inhibitory inputs jointly regulate postsynaptic current responses, enabling bidirectional modulation of synaptic activity. As schematically illustrated in Fig. 2a, this process can be mimicked in the proposed PbS/PEDOT:PSS/VO_*x*_ heterostructured device by using optical stimuli with different wavelengths as presynaptic inputs. In this all-optical synaptic configuration, near-infrared illumination serves as an excitatory stimulus to induce synaptic potentiation, whereas visible illumination acts as an inhibitory stimulus to trigger synaptic depression. Such wavelength-dependent bidirectional regulation provides the basis for all-optical synaptic weight modulation without electrical writing pulses.

To experimentally verify this wavelength-dependent bidirectional modulation, the postsynaptic current response was first examined under single optical pulse stimulation. As shown in Fig. 2b, a 980 nm pulse generates a positive excitatory postsynaptic current (EPSC), while a 525 nm pulse produces a negative inhibitory postsynaptic current (IPSC). After the optical stimulus is removed, both responses exhibit a gradual relaxation behavior, reflecting short-term memory characteristics analogous to those of biological synapses. To further verify the spectral selectivity of the device, additional wavelength-dependent measurements were performed. As shown in Fig. S5, near-infrared illumination at 940 and 980 nm induces positive photocurrent responses, with 980 nm producing a stronger potentiation effect. In contrast, visible illumination at 625, 525, and 465 nm induces negative photocurrent responses, and the inhibition amplitude increases at shorter wavelengths. These results confirm that near-infrared and visible light can selectively activate opposite conductance-modulation directions, supporting the selection of 980 and 525 nm as representative potentiating and inhibitory stimuli, respectively.

The optical sensitivity of the device was further evaluated by determining the minimum detectable optical power density. A criterion of ΔI_peak_ > 3σ_dark_ was adopted, where σ_dark_ represents the standard deviation of the dark-current noise. As shown in Fig. S6, reliable excitatory and inhibitory responses can still be detected under weak 980 and 525 nm illumination with power densities as low as 0.5 and 0.2 mW cm^−2^, respectively. This result demonstrates the high optical sensitivity of the proposed all-optical memristor and further supports its potential for low-power optical synaptic modulation.

In addition, the modulation amplitude of the postsynaptic current can be continuously regulated by optical stimulation parameters. As shown in Fig. 2c, under 980 nm illumination, the EPSC amplitude increases with irradiation time, and higher optical power density leads to a larger potentiation response. Conversely, under 525 nm illumination, the IPSC becomes progressively stronger with increasing irradiation time and light intensity. These results indicate that the synaptic weight of the device can be gradually and reversibly tuned by the wavelength, intensity, and duration of optical stimuli, providing a flexible optical programming strategy for analog weight modulation.

Short-term synaptic plasticity was further investigated using paired-pulse stimulation. Figure 2d shows the postsynaptic current responses triggered by two consecutive optical pulses. Under 980 nm stimulation, the second EPSC is larger than the first one, corresponding to paired-pulse facilitation (PPF). By contrast, under 525 nm stimulation, the second inhibitory response is enhanced relative to the first one, exhibiting paired-pulse depression (PPD)-like behavior. The dependence of the PPF/PPD index on the pulse interval is summarized in Fig. 2e. As the interval increases, the facilitation gradually weakens and the depression effect gradually recovers, reflecting the relaxation of internal state variables such as defect-state occupation and interfacial charge accumulation. These time-dependent relaxation characteristics are consistent with short-term synaptic memory behavior.

Beyond paired-pulse plasticity, the device also supports cumulative synaptic weight updating under pulse-train stimulation. As shown in Fig. 2f, successive 980 nm optical pulses induce a gradual increase in the postsynaptic current, and the potentiation amplitude becomes larger as the pulse number increases from 5 to 20. This pulse-number-dependent enhancement demonstrates the optical integration capability of the device. Frequency-dependent synaptic modulation was further examined using optical pulse trains with different repetition frequencies. As shown in Fig. 2g, increasing the repetition frequency of the optical pulse train enhances the postsynaptic current modulation, with 980 nm stimulation producing a larger positive ΔPSC and 525 nm stimulation producing a larger negative ΔPSC. These results indicate that the device can encode temporal input information through frequency-dependent all-optical synaptic weight updates.

The learning–forgetting–relearning behavior was further evaluated using a cyclic optical training protocol. As shown in Fig. 2h, repeated 980 nm optical pulses gradually increase the synaptic weight, corresponding to an optical learning process. After the optical stimulus is removed, the postsynaptic response spontaneously decays, indicating forgetting behavior. Notably, subsequent application of a small number of optical pulses can rapidly restore the synaptic weight to a high level, demonstrating relearning capability. This reversible training–relaxation–retraining process suggests that the device can emulate dynamic memory evolution under optical stimulation.

The temporal learning capability of the device was further examined by spike-timing-dependent plasticity (STDP) measurements. As shown in Fig. 2i, the synaptic weight change depends strongly on the relative timing difference between the potentiating and inhibitory optical stimuli. When the potentiating stimulus precedes the inhibitory stimulus, long-term potentiation (LTP) is obtained. Conversely, when the inhibitory stimulus precedes the potentiating stimulus, long-term depression (LTD) is induced. The resulting asymmetric STDP learning window indicates that the device can support timing-dependent synaptic learning rules using only optical stimuli.

The reproducibility of the all-optical synaptic response was evaluated across multiple devices from the same fabrication batch. Figures S7 and S8 summarize the EPSC and IPSC responses of 20 representative devices, respectively. The devices exhibit consistent response amplitudes and relaxation behaviors under identical optical stimulation, confirming good device-to-device uniformity for both near-infrared-induced potentiation and visible-light-induced inhibition. Furthermore, the array-level current mapping in Fig. S9 shows spatially uniform conductance modulation across the 32 × 32 memristor array, demonstrating the feasibility of extending the all-optical synaptic behavior from individual devices to integrated arrays.

The conductance potentiation and inhibition behaviors observed under different optical wavelengths can be attributed to the reversible modulation of oxygen-vacancy-related defect-state occupation in VO_*x*_. As an amorphous oxide semiconductor, VO_*x*_ contains abundant oxygen-vacancy-related defect states, which can modulate the effective carrier concentration and electrical conductivity. The band alignment and valence-band spectra provided in Figs. S10–S12 establish the energy-level landscape of the PbS/PEDOT:PSS/VO_*x*_ heterostructure. Based on these results, Fig. 2j summarizes the band alignment of the device and highlights the role of PEDOT:PSS as an electron-blocking and interfacial modulation layer. This heterostructure design enables a spectrally partitioned carrier-dynamics pathway: Near-infrared light mainly activates PbS-mediated electron generation and defect-state filling in VO_*x*_, whereas visible light mainly promotes VO_*x*_-related hole generation, interfacial recombination, and defect-state electron depletion. Therefore, the two opposite conductance-update directions are physically separated in the wavelength domain.

As illustrated in Fig. 2k, under 980 nm near-infrared illumination, PbS quantum dots act as the primary absorption layer and generate photogenerated electrons. The PEDOT:PSS interlayer regulates electron transfer across the PbS/VO_*x*_ interface and suppresses abrupt carrier injection. Consequently, photogenerated electrons are gradually transferred into oxygen-vacancy-related defect states in VO_*x*_. The progressive filling of these defect states increases the effective carrier concentration, resulting in continuous conductance potentiation. In contrast, as shown in Fig. 2l, under 525 nm visible-light illumination, optical absorption is mainly dominated by VO_*x*_. The generated holes accumulate near the PEDOT:PSS/VO_*x*_ interface, where they promote local recombination with electrons occupying oxygen-vacancy-related defect states. This process gradually depletes the defect-state electrons and weakens the conductive defect channels in VO_*x*_, resulting in visible-light-induced conductance suppression. Control experiments in Fig. S13 further support the important role of PEDOT:PSS-mediated interfacial processes in this suppression behavior.

In addition, PL measurements in Fig. S14 provide supporting evidence for interfacial carrier transfer. Taken together, these characterization results establish a consistent mechanism–property relationship. The absorption spectra and band alignment define the wavelength-selective excitation and carrier-transfer pathways; EPR and XPS reveal the existence and illumination-dependent modulation of oxygen-vacancy-related electronic states in VO_*x*_; and PL further verifies interfacial carrier transfer within the heterostructure. These processes eventually manifest as 980 nm-induced defect-state filling and conductance potentiation, as well as 525 nm-induced defect-state depletion and conductance suppression. Therefore, the observed LTP/LTD behavior is supported by a coherent chain of optical, interfacial, defect-state, and electrical evidence.

### Highly Linear and Energy-Efficient All-Optical Weight Update

The excellent linearity of the optical response exhibited by the device also originates from the defect-dominated gradual modulation mechanism described above. On the one hand, the PEDOT:PSS (AI 4083) interlayer introduces an interfacial barrier that effectively suppresses the instantaneous massive injection of photogenerated electrons, transforming the electron transfer into VO_*x*_ from an abrupt event into a smooth and controllable gradual process. On the other hand, the continuously distributed oxygen-vacancy defects in VO_*x*_ provide stepwise and tunable trapping channels for electrons, enabling the conductance modulation to evolve in a stable and nearly linear manner with increasing optical stimulation cycles.

**Fig. 3:**
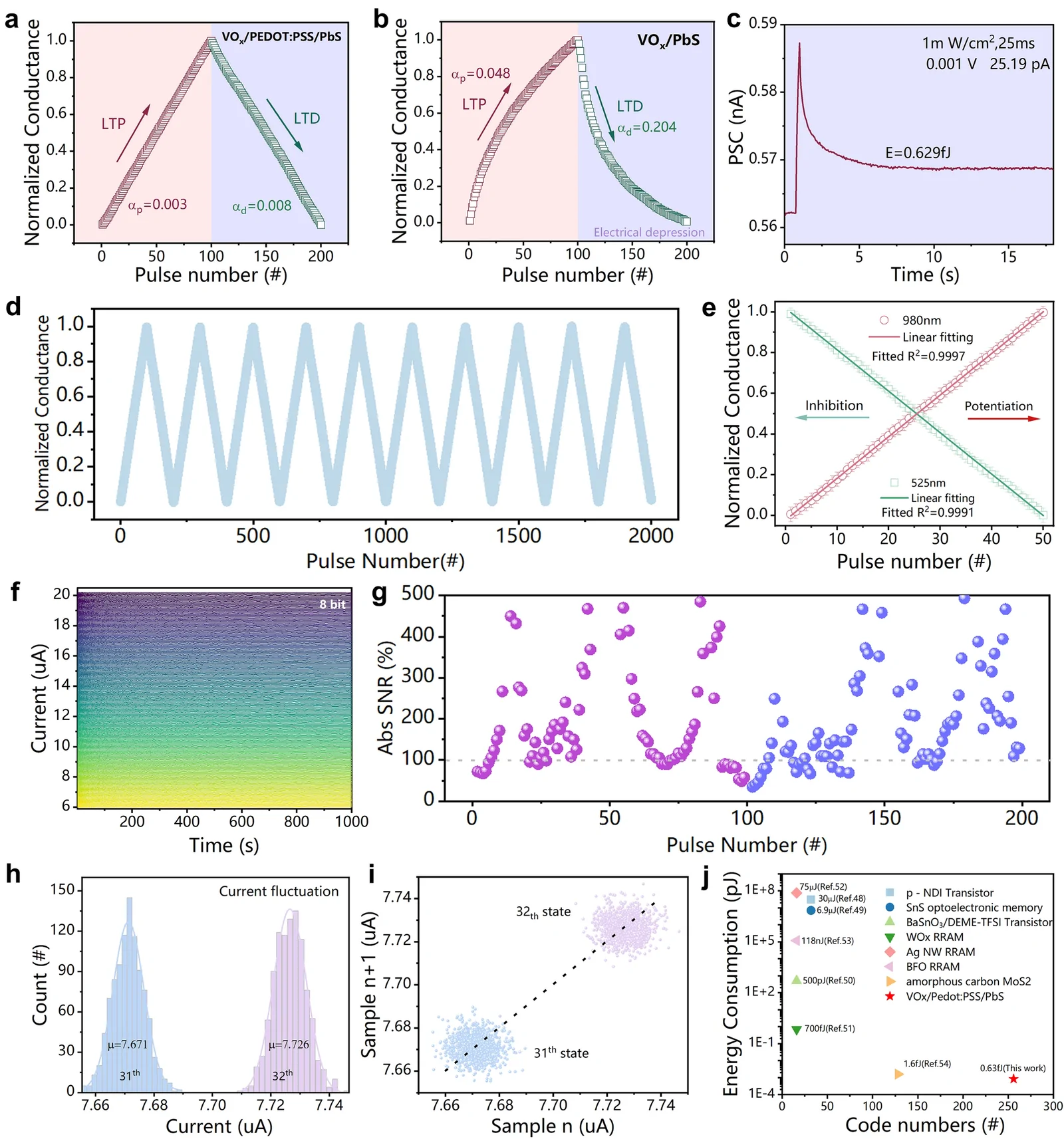
Highly linear and energy-efficient all-optical weight updates. **a**, **b** Weight-update linearity of devices with and without the PEDOT:PSS interlayer. **c** Transient photoresponse measured at a probing bias of 0.001 V, corresponding to an electrical energy consumption of 0.63 fJ per optically induced synaptic event. **d** Cyclic potentiation–depression behavior under repeated optical stimulation.** e** Linearity of normalized conductance evolution under 980 and 525 nm optical pulses. **f** 8-bit multilevel conductance states and retention characteristics. **g** SNR statistics of programmed weight states during LTP and LTD. **h**, **i** Current distribution and correlation analysis of adjacent weight states. **j** Benchmark comparison with previously reported optoelectronic synaptic and memristive devices [48–54]

As shown in Fig. 3a, the proposed PbS/PEDOT:PSS/VO_*x*_ device exhibits smooth and monotonic conductance modulation during both long-term potentiation (LTP) and long-term depression (LTD). Under near-infrared optical pulses, the normalized conductance gradually increases, whereas visible-light pulses induce a continuous and reversible conductance decrease. The extracted nonlinearity factors are as low as *α*_p_ = 0.003 and *α*_d_ = 0.008, demonstrating highly linear and symmetric bidirectional weight updates. In contrast, the control VO_*x*_/PbS device without the PEDOT:PSS interlayer shows much more nonlinear conductance evolution, with *α*ₚ = 0.048 and *α*_d_ = 0.204 (Fig. 3b). In this control device, the conductance change is mainly concentrated in the initial pulse region and rapidly approaches saturation, indicating uncontrolled carrier injection and poor depression linearity. These results confirm that the PEDOT:PSS interlayer is essential for moderating interfacial carrier-transfer kinetics and achieving highly linear all-optical weight modulation.

The influence of PEDOT:PSS thickness was further investigated to clarify the role of the interfacial regulation layer. As shown in Fig. S15, devices with PEDOT:PSS thicknesses of approximately 5, 15, and 25 nm exhibit clearly different LTP/LTD update characteristics. When the PEDOT:PSS layer is too thin, the interfacial carrier-regulation and charge-buffering effects are insufficient, leading to relatively nonlinear updates and premature conductance saturation. When the PEDOT:PSS layer is too thick, carrier transfer across the interface is overly suppressed, resulting in a reduced conductance modulation range and degraded weight-update controllability. The optimized thickness of approximately 15 nm provides the best balance between interfacial carrier regulation and efficient photoinduced charge transfer, yielding the most linear and reversible optical weight modulation. This thickness-dependent behavior further verifies that PEDOT:PSS functions as an active interfacial carrier-regulation layer rather than a passive spacer.

To examine whether the observed optical weight modulation is dominated by thermal effects, temperature-dependent measurements were performed. As shown in Fig. S16, the relaxation dynamics become faster as the temperature increases from 298.15 to 328.15 K. The fast relaxation time constant decreases from τ_1_ = 1.28 s at 298.15 K to τ_1_ = 0.52 s at 328.15 K, while the slow relaxation time constant decreases from τ_2_ = 6.49 s to τ_2_ = 5.64 s. This indicates that elevated temperature accelerates carrier relaxation, defect-state detrapping, and interfacial charge redistribution. Importantly, the bidirectional optical weight-update behavior remains stable over the tested temperature range. The LTP process maintains high linearity with fitted *R*^2^ values of 0.9997, 0.9994, and 0.9996 at 298.15, 313.15, and 328.15 K, respectively. The LTD process also remains highly linear, with fitted R^2^ values of 0.9991, 0.9987, and 0.9985. These results indicate that temperature mainly influences the relaxation kinetics but does not alter the switching polarity or significantly degrade the optical weight-update linearity, suggesting that the conductance modulation is governed primarily by wavelength-dependent carrier dynamics rather than a purely photothermal process.

The electrical energy associated with a single optically induced synaptic event was evaluated under an ultralow probing bias. As shown in Fig. 3c, under a probing bias of 0.001 V, a 25 ms optical pulse induces an incremental postsynaptic current variation of approximately 25.19 pA. The corresponding electrical energy consumption is calculated to be approximately 0.629 fJ per optically induced synaptic event. This value represents the incremental electrical energy associated with the transient optically induced response under the specified probing condition, rather than the total readout energy, optical programming energy, or system-level energy consumption. The optical programming energy and peripheral-circuit contributions are discussed separately in Note S4. The reversibility and cycling stability of optical weight modulation were then evaluated under repeated potentiation–depression operations. As shown in Fig. 3d, the device maintains highly reproducible conductance modulation over repeated optical pulse cycles, without obvious drift, collapse of the dynamic range, or cumulative degradation. This stable cycling behavior confirms the reversibility of the defect-state filling and depletion processes. In addition, the endurance test in Fig. S17 shows that the high- and low-conductance states remain stable over 1000 optical switching cycles, further demonstrating the robustness of the optical modulation process.

The linearity of the optical weight update was quantitatively examined by fitting the normalized conductance evolution as a function of pulse number. As shown in Fig. 3e, both the 980 nm-induced potentiation and the 525 nm-induced depression exhibit nearly ideal linear relationships with pulse number. The fitted *R*^2^ values reach 0.9997 for potentiation and 0.9991 for depression, indicating highly predictable and controllable bidirectional optical weight writing. Such linear weight modulation is particularly important for hardware neural networks, because nonlinear and asymmetric conductance updates usually lead to accumulated training errors and degraded inference accuracy. The device-to-device weight-update characteristics shown in Fig. S18 further reveal consistent potentiation–depression behavior among representative devices, confirming good reproducibility in fine optical weight control.

In addition to high linearity, the device also supports high-resolution multilevel conductance programming. As shown in Fig. 3f, 256 gradually programmable conductance states are achieved through optical pulse programming, corresponding to an effective 8-bit weight resolution. The programmed states remain stable during the measurement window, indicating good short-term stability of multilevel optical weights. The raw 8-bit programming trace is provided in Fig. S19, further confirming continuous and high-resolution conductance modulation. Long-term retention measurements in Fig. S20 show that representative conductance states, including the 1st, 30th, 60th, 90th, 120th, 150th, 180th, 210th, and 240th states, remain clearly separated over 10^4^ s. Moreover, Fig. S21 confirms that the 8-bit conductance states can be repeatedly programmed over multiple training cycles, demonstrating good multilevel stability during repeated optical weight updates.

To evaluate the reliability of the programmed conductance states, the signal-to-noise ratio (SNR) of each optical weight state was analyzed. As shown in Fig. 3g, most programmed states maintain an absolute SNR above the reliable-readout threshold, indicating that the conductance levels can be distinguished against intrinsic current fluctuations. Specifically, more than 78% of the states during the LTP process and more than 81% of the states during the LTD process satisfy SNR > 1. This result confirms that the majority of optically programmed weight states can be reliably read and used for neuromorphic computation. Besides, the distinguishability of adjacent conductance states was further examined using the 31st and 32nd programmed states. As shown in Fig. 3h, the current distributions of these two adjacent states are clearly separated, indicating low overlap and good state discriminability. The state-correlation analysis in Fig. 3i further shows a strong linear relationship between adjacent-state measurements, confirming the stability and reproducibility of fine conductance tuning. These results demonstrate that the proposed device can support reliable high-resolution analog weight storage.

Finally, the energy consumption and coding capability of the proposed device were compared with those of previously reported optoelectronic synaptic and memristive devices. As summarized in Fig. 3j, the PbS/PEDOT:PSS/VO_*x*_ all-optically programmable memristor achieves an electrical energy consumption of 0.63 fJ per optically induced synaptic event under an ultralow probing bias, together with an effective 8-bit conductance resolution and highly linear optical weight modulation. Compared with representative optoelectronic synaptic devices, the proposed device offers a favorable combination of low event-level electrical energy consumption, high conductance-state density, and excellent update linearity. These characteristics make it a promising building block for energy-efficient and high-precision neuromorphic hardware.

### In-Memory Convolution Enabled by All-Optical Synaptic Arrays

**Fig. 4:**
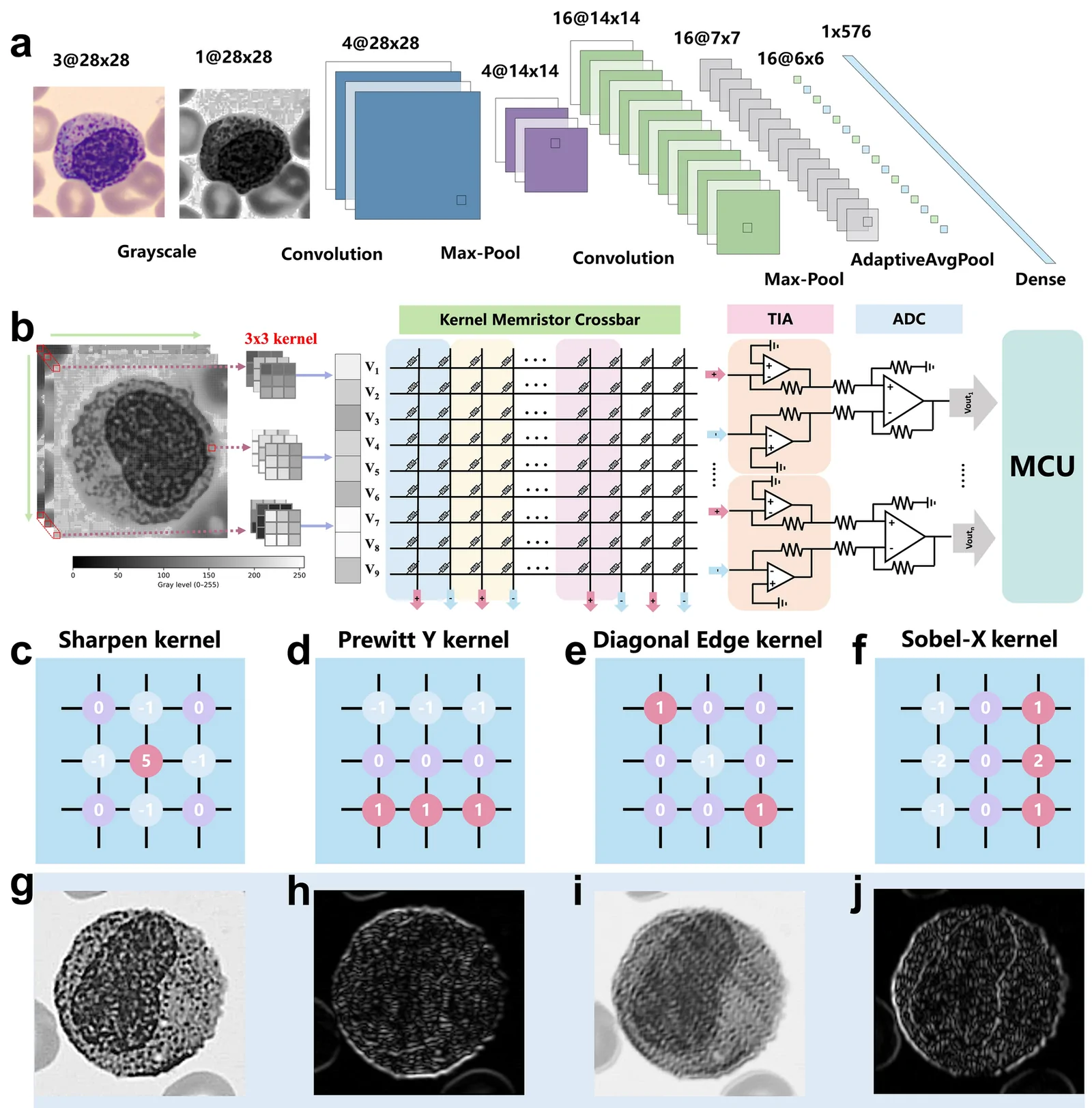
In-memory convolution enabled by all-optical synaptic arrays.** a** Schematic of the in-memory convolution process, where input images are mapped onto the memristor crossbar array using a sliding-window scheme. **b** Hardware architecture of the convolution module, including the memristor array, transimpedance amplifiers (TIAs), analog-to-digital converters (ADCs), and microcontroller unit (MCU). **c**–**f** Representative convolution kernels (sharpening, Prewitt-Y, diagonal edge detection, and Sobel-X) implemented using the all-optical synaptic devices. **g–j** Corresponding hardware-computed output images

Building on these results, we further constructed a convolutional neural network (CNN)-like hardware convolution module using the proposed all-optical synaptic devices, and employed microscopic cell images from the BloodMNIST dataset as inputs to evaluate its computational capability for neuromorphic visual feature extraction. As illustrated in Fig. 4a, the input cell microscopy images are first preprocessed and mapped into grayscale matrices, which are then fed into the memristor crossbar array in a sliding-window manner to perform convolution operations directly within the memory (in-memory computing). The convolution-kernel weights are encoded by the multilevel conductance states of the devices and mapped onto the memristor crossbar in the form of a 3 × 3 weight matrix, enabling parallel analog multiply–accumulate (MAC) operations.

As shown in Fig. 4b, the constructed memristor crossbar array functions as a convolution-kernel array and operates in conjunction with peripheral transimpedance amplifiers (TIAs) and analog-to-digital converters (ADCs). The output currents from the array are converted into digital signals and transmitted to a microcontroller unit (MCU), thereby completing the full hardware computation pipeline from image input to feature extraction. This architecture fully exploits the intrinsic advantages of memristors in the co-localization of weight storage and computation, effectively mitigating the frequent data movement inherent to conventional von Neumann architectures. To demonstrate the computational capability of the system under representative visual operators, Fig. 4c–f presents the convolution kernels implemented using the proposed devices—including sharpening, Prewitt-Y, diagonal edge detection, and Sobel-X filters—while Fig. 4g–j shows their corresponding hardware-computed output images. The resulting images clearly extract texture, edge, and directional features from the cell images under different operators and show good agreement with the theoretical convolution results. These observations validate the effectiveness and reliability of multilevel weight encoding and all-optical synaptic modulation for analog convolution operations. Therefore, these results indicate that the proposed all-optical synaptic devices not only exhibit high linearity, low energy consumption, and good uniformity at the device level, but also support programmable, multi-operator parallel convolution at the system level, providing a solid foundation for the development of energy-efficient and array-integrated brain-inspired visual processing hardware.

### End-to-End Neuromorphic Inference Based on All-Optical Synapses

**Fig. 5:**
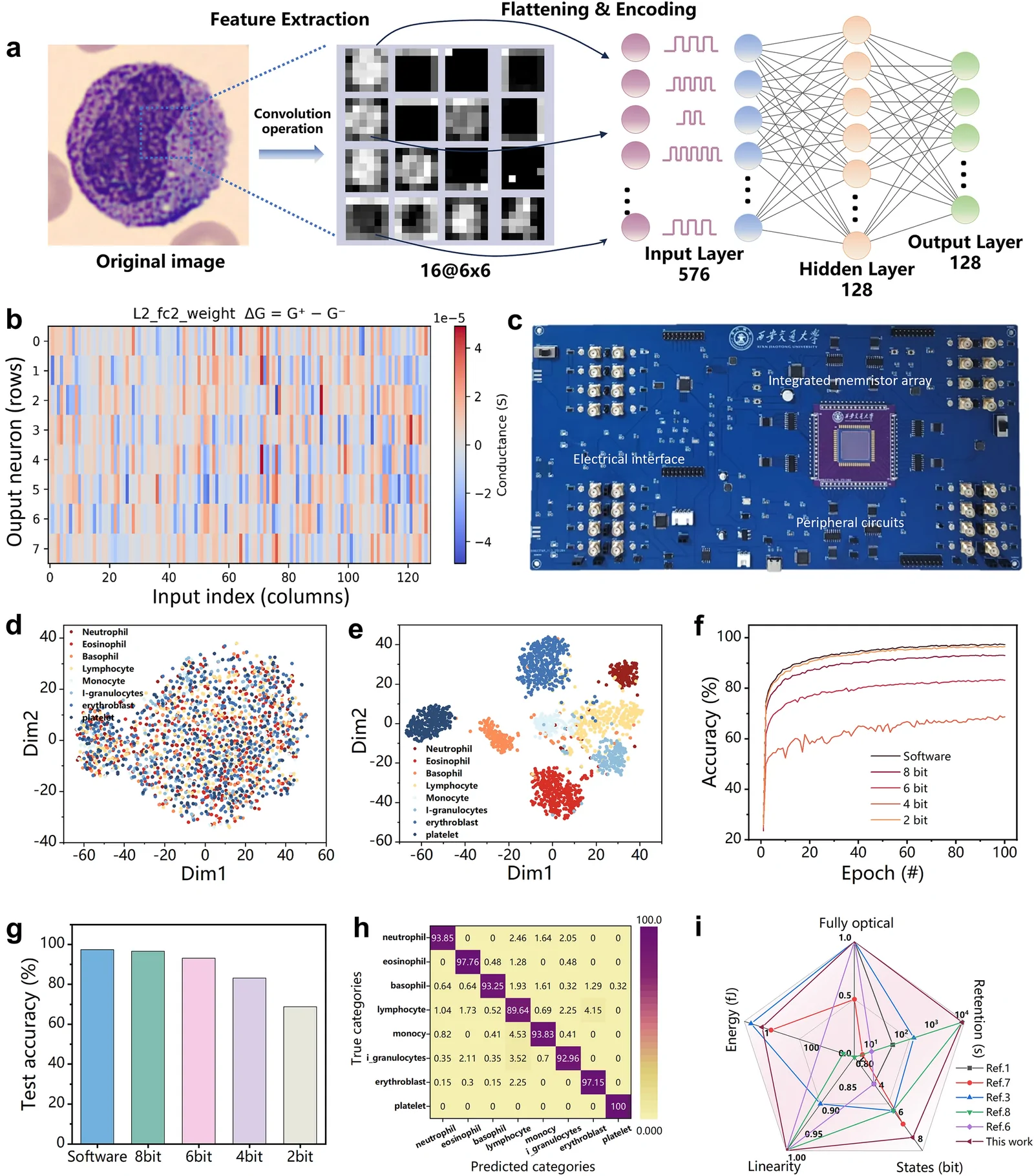
End-to-end neuromorphic inference enabled by all-optical synaptic arrays. **a** Schematic illustration of the end-to-end inference framework, including convolutional feature extraction and fully connected classification. **b** Differential conductance mapping (ΔG = G^+^ − G^−^) of synaptic weights for the final fully connected layer. **c** Photograph of the neuromorphic computing hardware platform. **d, e** Visualization of network output features in reduced-dimensional space, showing clear class separation after training. **f** Training and inference performance under different weight quantization precisions. **g** Test accuracy statistics for different quantization bit widths. **h** Confusion matrix obtained under 8-bit weight precision. **i** Comparison of energy consumption, weight resolution, and linearity with previously reported optoelectronic synaptic and memristive neuromorphic systems

Building on the convolutional feature extraction results, we further constructed an end-to-end neural network inference framework supported by the all-optical synaptic devices. In this framework, the convolutional features are mapped to fully connected layers to generate classification outputs, enabling a systematic evaluation of the device performance in practical neuromorphic inference tasks. As illustrated in Fig. 5a, BloodMNIST images are first partitioned into local patches and converted into input pulse sequences. These inputs are processed through a neural network architecture in which the trained synaptic weights are mapped onto the experimentally accessible conductance states of the memristor array. In this framework, the all-optical synaptic device serves as a hardware weight unit, enabling image features to be transformed into classification outputs through array-level vector–matrix operations.

To implement signed neural network weights in hardware, a differential conductance mapping scheme was adopted, where each weight is represented by the conductance difference between two devices, ΔG = G^+^ − G⁻. As shown in Fig. 5b, the weight distribution of the final fully connected layer is mapped into positive and negative conductance components, enabling bipolar weight representation using optically programmed conductance states. Figure S22 further presents the conductance maps corresponding to the two fully connected layers, FC1 and FC2, together with their weight distributions before quantization. These results indicate that the trained network weights can be accommodated within the available conductance window of the device, supporting the feasibility of hardware mapping for neural network inference. It should be noted that the present prototype adopts an ex situ training and hardware inference strategy: The network weights are first optimized in software and subsequently mapped onto the memristor array for hardware-level inference. The highly linear and multilevel all-optical conductance modulation demonstrated above provides a device-level foundation for future in situ optical learning.

Figure 5c shows a photograph of the constructed neuromorphic hardware platform, which integrates the memristor array with peripheral addressing and readout circuits. The integrated array module and enlarged optical image of a representative local cross-point region are shown in Fig. S23, confirming the physical addressability of the 32 × 32 crossbar array. To clarify the optical coupling geometry, Fig. S24 illustrates the focused-light illumination scheme used for optical programming. In this configuration, the incident light is locally delivered to the exposed or edge active region around the selected cross-point, rather than relying on direct transmission through a thick metal electrode. This local optical-access geometry enables targeted optical modulation of selected devices while reducing unintended illumination of neighboring regions.

The detailed optical programming and electrical-readout architecture is illustrated in Fig. S25. The platform consists of FPGA-based control logic, DAC-driven word-line addressing, multiplexers, the 32 × 32 memristor crossbar array, a DMD-based optical addressing module, transimpedance amplification, and ADC-based readout. During optical programming, the DMD projects patterned illumination onto the selected cell or selected array region, while the surrounding DMD pixels are kept in the off state to minimize unintended illumination of neighboring cells. This spatially defined optical addressing enables localized weight programming without applying electrical write pulses to half-selected cells, thereby helping to reduce electrically induced write disturbance and half-selected-cell interference. During electrical readout, the conductance states are addressed through FPGA-controlled row/column multiplexing and peripheral readout circuits. The output currents are converted into voltage signals by TIAs and subsequently digitized by ADCs for neural network inference.

The possible selected and distributed sneak-current paths in the crossbar array are schematically illustrated in Fig. S26. To quantitatively evaluate array-level parasitic leakage, we further performed selected/unselected current measurements, half-selected-cell leakage analysis, read-margin extraction, read-voltage-dependent characterization, and a simplified 32 × 32 resistive-network SPICE simulation, as summarized in Figs. S27–S31. The results show that parasitic leakage is measurable and increases as the background conductance changes from HRS to LRS. Nevertheless, under the tested operating conditions, the selected-cell signal remains substantially larger than the maximum measured unselected current. Even under the most unfavorable LRS background, the selected-to-maximum-unselected current ratio remains above 30, while the normalized current margin remains above 96%. These results support reliable readout in the present 32 × 32 prototype array. For larger passive arrays, additional leakage-suppression strategies, such as selector integration, 1T1R or 1S1R architectures, self-rectifying cells, improved optical addressing accuracy, and dedicated V/2 or V/3 biasing schemes, will still be required.

To evaluate the discriminative capability of the network, the feature distributions were visualized in reduced-dimensional spaces. As shown in Fig. 5d, before effective feature learning, different BloodMNIST cell categories are strongly mixed in the projected feature space. After training and hardware-mapped weight representation, the samples form more clearly separated clusters, as shown in Fig. 5e. This improved feature separability indicates that the optically programmed synaptic weights can support effective representation and classification of high-dimensional biomedical image features. In particular, the influence of weight quantization precision on inference performance was then systematically evaluated. As shown in Fig. 5f, the floating-point software model shows the fastest convergence and highest accuracy, while reducing the weight precision gradually degrades both convergence speed and final classification performance. Nevertheless, the 8-bit hardware-mapped network closely approaches the software baseline, indicating that the 8-bit conductance resolution of the device is sufficient to preserve most of the trained weight information. The corresponding test accuracy statistics are summarized in Fig. 5g. The 8-bit hardware-mapped inference achieves a test accuracy of 96.5%, which remains close to the floating-point software result and is clearly higher than those obtained with lower bit-width mappings. This result highlights the importance of highly linear optical weight updates and multilevel conductance resolution for reducing quantization loss and maintaining inference accuracy in neuromorphic hardware.

The classification reliability of the 8-bit mapped network was further examined using the confusion matrix shown in Fig. 5h. High recognition accuracy is achieved across different BloodMNIST categories, indicating that the proposed optically programmed neuromorphic hardware platform can support robust multiclass biomedical image classification. Although direct comparison with previously reported neuromorphic systems can be influenced by differences in datasets, network architectures, and hardware implementations, the achieved BloodMNIST inference performance demonstrates the practical potential of the proposed all-optical synaptic device under the same task and mapping framework. Finally, Fig. 5i compares the proposed device with representative optoelectronic synaptic and memristive neuromorphic systems in terms of energy consumption, weight-state number, linearity, retention, and all-optical programming capability. The PbS/PEDOT:PSS/VO_*x*_ all-optically programmable memristor simultaneously offers an electrical energy consumption of 0.63 fJ per optically induced synaptic event, an effective 8-bit conductance resolution, a retention time of 104 s, highly linear bidirectional weight updates, and fully optical synaptic programming. These combined advantages highlight its potential as an energy-efficient and array-compatible hardware building block for high-precision neuromorphic inference.

## Conclusions

In summary, we have demonstrated a spectrally partitioned, all-optically programmable memristive device based on a PbS/PEDOT:PSS/VO_*x*_ heterostructure. Through wavelength-selective carrier dynamics, near-infrared, and visible light respectively induce conductance potentiation and depression within a single device, eliminating the need for electrical write signals during weight programming. The PEDOT:PSS interlayer regulates interfacial electron transfer and suppresses abrupt carrier injection, while the continuously distributed oxygen-vacancy-related defect states in VO_*x*_ enable gradual and reversible conductance modulation. This coordinated heterostructure design results in highly linear and symmetric bidirectional weight updates, an effective 8-bit conductance resolution, and an ultralow electrical energy consumption of 0.63 fJ per optically induced synaptic event. Furthermore, the devices were integrated into a 32 × 32 all-optically programmed memristor-array prototype for hardware-based BloodMNIST microscopic blood-cell image classification, achieving an accuracy of 96.5% with strong robustness against noise perturbations. The array-level demonstration supports the feasibility of extending the wavelength-selective optical weight-modulation mechanism from individual devices to integrated neuromorphic hardware under the tested operating conditions. Overall, this work provides a viable material and device strategy for developing high-linearity, low-power, and spectrally programmable neuromorphic vision systems.

## Supplementary Information

Below is the link to the electronic supplementary material.Supplementary file1 (DOCX 9528 kb)
