# Supplementary material for: Spectrally Partitioned All-Optical Memristors for Highly Linear Neuromorphic Vision Systems
Source: Nanomicro Lett. 2026 Aug 31;19:42. doi: 10.1007/s40820-026-02331-4 (PMC13529625; doi:10.1007/s40820-026-02331-4)
Supplement: Supplementary file 1 [file 40820_2026_2331_MOESM1_ESM.docx]

Supporting Information for

**Spectrally Partitioned All-Optical Memristors for Highly Linear Neuromorphic Vision Systems**

Junchao Zhang ^1,2^, Bai Sun ^1,2,^*, Songling Wang ^3^, Guangdong Zhou ^4^, Linfeng Sun ^5^, Zelin Cao ^1,2^, Kaikai Gao ^1,2^, Xiaojun Li ^1,2^, Xiaoliang Chen ^1,2^, Jinyou Shao ^1,2,^*

^1^ Frontier Institute of Science and Technology, and Interdisciplinary Research Center of Frontier Science and Technology, Xi'an Jiaotong University, Xi'an, Shaanxi 710049, P. R. China

^2^ Micro-and Nano-technology Research Center, State Key Laboratory for Manufacturing Systems Engineering, Xi’an Jiaotong University, Xi’an, Shaanxi 710049, P. R. China

^3^ State Key Laboratory of Structural Chemistry, Fujian Institute of Research on the Structure of Matter, Chinese Academy of Sciences, Fuzhou 350002, P. R. China

^4^ College of Artificial Intelligence, Brain-inspired Computing & Intelligent Control of Chongqing Key Lab, Southwest University, Chongqing 400715, P. R. China

^5^ Centre for Quantum Physics, Key Laboratory of Advanced Optoelectronic Quantum Architecture and Measurement (MOE), School of Physics, Beijing Institute of Technology, Beijing 100081, P. R. China

*** Corresponding authors. E-mail: [baisun@xjtu.edu.cn](mailto:baisun@xjtu.edu.cn) (Bai Sun); [jyshao@xjtu.edu.cn](mailto:jyshao@xjtu.edu.cn) (Jinyou Shao)

**S1 Supplementary Text**

***Note S1 Phenomenological rate-equation model for wavelength-dependent carrier dynamics***

To provide a quantitative description of the wavelength-dependent potentiation and depression dynamics, a phenomenological rate-equation model was introduced to describe the evolution of oxygen-vacancy-related conductive defect-state occupation under optical stimulation. The normalized occupation of conductive defect states, n_d_ , is expressed as:

$$\frac{dn_{d}}{d_{t}}=k_{NIR}\left( 1-n_{d} \right)-k_{vis}n_{d}-\frac{n_{d}-n_{d,0}}{\tau_{relax}}$$

where $k_{NIR}$ represents the near-infrared-induced electron injection rate into oxygen-vacancy-related defect states, $k_{vis}$ denotes the visible-light-induced recombination/de-occupation rate, $\tau_{relax}$ is the defect-state relaxation time constant after removal of illumination, and $n_{d,0}$ is the equilibrium defect-state occupation in the dark state. The visible-light-induced recombination/de-occupation term phenomenologically represents trap-assisted electron–hole recombination, consistent with the Shockley–Read–Hall framework [S9, S10]. Related photoinduced carrier trapping and redistribution processes have also been reported to induce negative photoconductance in optoelectronic heterostructures [S11].

Under 980 nm illumination, the electron-injection term dominates, and the defect-state occupation gradually increases toward a higher steady-state value:

$$n_{d}\left( t \right)=n_{d,\infty}^{NIR}-(n_{d,\infty}^{NIR}-n_{d}(0))exp(-k_{NIR}t)$$

This progressive filling of conductive defect states corresponds to continuous conductance potentiation. Under 525 nm illumination, the recombination/de-occupation term dominates, and the occupation of conductive defect states decreases gradually:

$$n_{d}\left( t \right)=n_{d,\infty}^{vis}+(n_{d}\left( 0 \right)-n_{d,\infty}^{vis})exp(-k_{vis}t)$$

where $n_{d,\infty}^{vis}$ is lower than the initial defect-state occupation under visible-light-induced recombination. This process describes the gradual depletion of conductive defect states and the corresponding conductance depression behavior. Assuming that the device conductance is approximately proportional to the occupation of conductive defect states, the conductance evolution can be expressed as:

$$G\left( t \right)=G_{base}+\alpha n_{d}(t)$$

where $G_{base}$ is the baseline conductance and α is the contribution coefficient of defect-state occupation to the device conductance. Within the experimentally used optical-pulse range, the gradual evolution of n_d_ gives rise to smooth and quasi-linear conductance modulation, consistent with the measured potentiation and depression characteristics.

***Note S2 Quantification of weight-update nonlinearity***

To quantitatively evaluate the nonlinearity of the optical weight-update process, the conductance evolution during potentiation (LTP) and depression (LTD) was analyzed using a standard exponential fitting approach widely adopted for memristive synaptic devices. The measured conductance values G(n) were first normalized into the range of 0-1 according to:

$$G_{norm}\left( n \right)=\frac{G(n)-G_{min}}{G_{max}-G_{min}}$$

where G_min_ and G_max_ represent the minimum and maximum conductance states within one complete update cycle, respectively. For the potentiation (LTP) process, the normalized conductance evolution was fitted using:

$$G_{p}\left( n \right)=\frac{1-exp(-\alpha_{p}n)}{1-exp(-\alpha_{p}N)}$$

For the depression (LTD) process, the conductance was first transformed into an increasing form:

$$G_{D}^{*}\left( n \right)=\frac{G_{max}-G(n)}{G_{max}-G_{min}}$$

and then fitted using the same expression:

$$G_{D}^{*}\left( n \right)=\frac{1-exp(-\alpha_{d}n)}{1-exp(-\alpha_{d}N)}$$

In these equations, n denotes the pulse number, N is the total number of applied pulses, and α_p_ and α_d_ correspond to the nonlinearity factors for the potentiation and depression processes, respectively. The fitting parameters were extracted by least-squares fitting of the experimental data. The LTP and LTD processes were treated independently, and pulse numbers were re-indexed from n = 0 to N.

The parameter α reflects the deviation of the conductance evolution from an ideal linear update. A larger α value corresponds to stronger exponential saturation behavior, whereas a smaller α value indicates a more gradual and nearly linear conductance evolution. In the limit of α → 0, the model approaches an ideal linear relationship.

Therefore, smaller α_p_ and α_d_ values indicate improved linearity and more uniform weight-update characteristics, which are desirable for accurate and stable neuromorphic computing.

***Note S3*** ***Estimated latency and throughput of the hardware inference system***

To further evaluate the practical system-level performance of the optically programmable neuromorphic hardware prototype, we estimated the inference latency and throughput of the present hardware platform. It should be noted that the optical programming process in this work is used for off-line weight configuration and is therefore not included in the inference latency. The latency discussed here refers only to the hardware inference stage, including array addressing, electrical readout, signal conversion, and digital processing.

As described in the main text, the current system is built around a 32 × 32 memristive crossbar array integrated with FPGA-based control logic, DACs, word-line drivers, multiplexers, transimpedance amplifiers (TIAs), and a 10-bit ADC. During inference, the array output current is converted into a voltage signal by the TIA, digitized by the ADC, and subsequently processed by the control unit. Therefore, the total inference latency can be approximately expressed as:

$$T_{inf}\approx N_{read}\times\left( t_{addr}+t_{settle}+t_{ADC} \right)+t_{digital}$$

where $N_{read}$ is the number of array read operations required for one image, $t_{addr}$ is the delay associated with array addressing and multiplexing, $t_{settle}$ is the settling time of the transimpedance amplifier, $t_{ADC}$ is the ADC conversion time, and $t_{digital}$ is the overhead of FPGA/MCU-based digital processing.

For the BloodMNIST classification task, the convolutional preprocessing is performed using a 3 × 3 sliding-window scheme. Assuming a 28 × 28 input image and unit stride without padding, one convolution kernel corresponds to approximately:

$$\left( 28-3+1 \right)\times\left( 28-3+1 \right)=676$$

read positions. For multiple convolution kernels, the total number of read cycles is correspondingly increased to the order of 10^3^ per image. Considering that the present system is a PCB-level prototype rather than a fully monolithically integrated chip, a conservative peripheral readout time of 10-50 μs per read cycle was adopted for order-of-magnitude estimation. Under this assumption, the system-level inference latency is estimated to be on the order of approximately 10-100 ms per image, depending on the number of convolution kernels and the timing overhead of peripheral circuits. Accordingly, the throughput is estimated to be on the order of approximately 10-100 images s^-1^.

This estimation indicates that the present hardware platform supports practical prototype-level hardware inference. Moreover, since the dominant delay mainly originates from peripheral circuit operations rather than the intrinsic response of the memristive device, the latency and throughput could be further improved by optimizing array-parallel readout, analog front-end design, ADC conversion, and on-chip signal-processing integration.

***Note S4 Energy consumption analysis***

To provide a more complete evaluation of the energy characteristics of the all-optically programmable memristive hardware platform, the energy consumption is discussed from three aspects: the incremental electrical energy associated with an optically induced synaptic event, the incident optical programming energy, and the system-level peripheral energy. For the transient low-current photoresponse shown in Fig. 3c, the event-level electrical energy was calculated as:

$$E_{\mathrm{event}}=V_{\mathrm{probe}}\times{\Delta I}_{\mathrm{psc}}\times t_{\mathrm{pulse}}$$

where V_probe_ is the probing bias, ΔI_PSC_ is the photoinduced incremental postsynaptic-current variation, and t_pulse_ is the optical pulse width. Under a probing bias of 0.001 V, a 25 ms optical pulse induces an incremental current variation of approximately 25.19 pA, corresponding to an electrical energy consumption of approximately 0.629 fJ per optically induced synaptic event. This value represents the incremental electrical energy associated with the transient optically induced response under the specified probing condition, rather than the total readout energy, optical programming energy, or system-level energy consumption. For optical weight programming, the incident optical energy delivered to the selected device or array region can be estimated as:

$$E_{\mathrm{opt}}=P_{\mathrm{density}}\times A_{illum}\times t_{\mathrm{pulse}}$$

where P_density_ is the calibrated optical power density at the sample plane, A_illum_ is the illuminated area, and t_pulse_ is the optical pulse duration. In the array-level measurements, the representative illuminated cross-point region is approximately 100 μm × 100 μm, corresponding to 1 × 10^-4^ cm^2^. With an optical pulse width of 0.15 s, the incident optical programming energy is estimated to be approximately 153 nJ per pulse for 980 nm illumination at 10.2 mW cm^-2^ and approximately 69 nJ per pulse for 525 nm illumination at 4.6 mW cm^-2^.

These optical energy values refer to the energy incident on the selected device or array region at the sample plane. In a practical hardware system, the total energy budget also depends on the external light source, DMD projection module, FPGA, DAC/ADC modules, multiplexers, transimpedance amplifiers, and control electronics. The peripheral energy can generally be expressed as:

$$E_{peri}=\sum_{i} p_{i}\times t_{i}$$

where P_i_ and t_i_ are the operating power and active time of each peripheral module, respectively. Since the present system is a PCB-level prototype using external optical and electronic modules, the wall-plug energy of these peripheral components depends strongly on the specific hardware implementation, optical coupling efficiency, readout parallelism, and operation duty cycle. Therefore, the reported 0.63 fJ value reflects the incremental electrical energy associated with a single optically induced synaptic event under the specified probing condition, while the optical programming energy and peripheral energy are separately estimated and discussed. This distinction provides a more complete and accurate assessment of the energy characteristics of the present all-optical memristive hardware platform.

**S2 Supplementary Figures and Tables**

**Fig. S1** Cross-sectional EDS line-scan profile of the PbS/PEDOT:PSS/VOₓ/Au/Al/SiO_2_/Si multilayer structure.

**Fig. S2** Structural and size characterization of PbS quantum dots. **a** Low-magnification transmission electron microscopy (TEM) image of PbS quantum dots, showing a relatively uniform dispersion without obvious large-scale aggregation. **b** High-resolution TEM (HRTEM) image of PbS quantum dots. **c** Particle-size distribution histogram extracted from TEM images, indicating a narrow size distribution mainly centered at approximately 4-6 nm. **d** Enlarged HRTEM image from the marked region in panel b, showing distinct lattice fringes and confirming the crystalline nature of the PbS quantum dots.

**Fig. S3** AFM characterization of VOₓ and VOₓ/PEDOT:PSS/PbS films. **a** Three-dimensional AFM topography image of the VOₓ thin film. **b** Two-dimensional AFM height image of the VOₓ thin film. **c** Height profile extracted along the dashed line in panel b, showing an RMS roughness of approximately 7.26 nm. **d** Three-dimensional AFM topography image of the VOₓ/PEDOT:PSS/PbS film. **e** Two-dimensional AFM height image of the VOₓ/PEDOT:PSS/PbS film. **f** Height profile extracted along the dashed line in panel e, showing a reduced RMS roughness of approximately 3.78 nm.

**Fig. S4** X-ray photoelectron spectroscopy characterization of PEDOT:PSS/PbS and VOₓ films. **a** XPS survey spectrum of the PEDOT:PSS/PbS composite film, showing the characteristic C 1s, O 1s, S 2p, and Pb 4f signals. **b** High-resolution V 2p XPS spectrum of the pristine VOₓ film measured under dark conditions, deconvoluted into V^4+^ and V^5+^ components. The coexistence of V^4+^/V^5+^ states indicates the non-stoichiometric nature of the VOₓ film and the presence of oxygen-vacancy-related electronic states.

**Fig. S5** Wavelength-dependent optical responses of the PbS/PEDOT:PSS/VOₓ device. **a** Transient current changes under near-infrared illumination at 940 and 980 nm. **b** Transient current changes under visible illumination at 625, 525, and 465 nm.

**Fig. S6** Minimum detectable optical power for reliable optical switching. **a** Positive current response induced by a 980 nm optical pulse with a power density of 0.5 mW cm⁻² and a pulse width of 1 s. **b** Negative current response induced by a 525 nm optical pulse with a power density of 0.2 mW cm^-2^ and a pulse width of 1 s.

**Fig. S7** Device-to-device uniformity of optically induced EPSC responses. **a** Transient excitatory postsynaptic current (EPSC) responses measured from five representative devices under identical optical pulse stimulation. **b** Statistical distribution of EPSC peak currents extracted from 20 devices, showing a small device-to-device variation with a coefficient of variation (σ/μ) of approximately 1.61%.

**Fig. S8** Device-to-device uniformity of optically induced IPSC responses. **a** Transient inhibitory postsynaptic current (IPSC) responses measured from five representative devices under identical visible-light pulse stimulation. **b** Statistical distribution of IPSC peak currents extracted from 20 devices, showing a small device-to-device variation with a coefficient of variation (σ/μ) of approximately 1.97%.

**Fig. S9** Spatial distribution of array current states before and after optical programming. **a** Two-dimensional current map of the 32 × 32 memristor array in the initial state before optical stimulation. **b** Two-dimensional current map of the array after 980 nm optical programming, showing an overall increase in the readout current across the array.

**Fig. S10** Optical bandgap extraction of individual functional layers. **a** Tauc plot of $\left( \alpha h\nu)^{2} \right.$ versus photon energy$\left( h\nu\right)$ for the PbS quantum-dot film, indicating an optical bandgap of approximately 1.17 eV. **b** Tauc plot of the PEDOT:PSS (AI 4083) film, showing a wide optical bandgap of approximately 3.65 eV. **c** Tauc plot of the VOₓ thin film, yielding an optical bandgap of approximately 2.28 eV. The optical bandgaps were extracted by linear fitting of the absorption edge and extrapolation to the energy axis.

**Fig. S11** Valence band spectra of individual functional layers. **a** Valence band XPS spectrum of the PbS quantum-dot film, with the valence band maximum (VBM) extracted as approximately 0.87 eV below the Fermi level. **b** Valence band XPS spectrum of the PEDOT:PSS (AI 4083) film, yielding a VBM of approximately 0.71 eV. **c** Valence band XPS spectrum of the VOₓ thin film, showing a VBM of approximately 2.09 eV. The dashed lines indicate linear extrapolation of the valence band onset used to determine the VBM positions.

**Fig. S12** Energy band alignment of the PbS/PEDOT:PSS/VOₓ heterostructure. **a** Schematic energy-level diagrams of PbS quantum dots, PEDOT:PSS (AI 4083), and VOₓ thin films, constructed from the optical bandgaps obtained by Tauc analysis and the valence band maxima extracted from XPS measurements. **b** Energy band alignment of the PbS/PEDOT:PSS/VOₓ heterostructure, illustrating the electron-blocking layer (EBL) formed by PEDOT:PSS and the built-in electric field (E_b_) across the heterointerfaces.

**Fig. S13** Control experiment on VOₓ/PbS devices without the PEDOT:PSS interlayer. **a** Normalized transient current response of the VOₓ/PbS device under 980 nm near-infrared illumination, showing obvious photocurrent enhancement and subsequent relaxation after light-off. **b** Normalized transient current response under 525 nm visible-light illumination, showing no obvious inhibitory current response. The shaded regions indicate the illumination duration.

**Fig. S14** Photoluminescence quenching of PbS in the PbS/PEDOT:PSS/VOₓ heterostructure. Photoluminescence (PL) spectra of pristine PbS quantum dots and the PbS/PEDOT:PSS/VOₓ heterostructure measured under 800 nm excitation. The strongly suppressed PL intensity in the heterostructure indicates reduced radiative recombination of photoexcited carriers in PbS.

**Fig. S15** Thickness-dependent all-optical weight-update characteristics of devices with different PEDOT:PSS interlayer thicknesses. **a-c** Normalized conductance evolution during optical potentiation and depression for devices with PEDOT:PSS interlayer thicknesses of approximately 5 nm (a), 15 nm (b), and 25 nm (c), respectively. The extracted nonlinearity factors show that the device with an optimized PEDOT:PSS thickness of approximately 15 nm exhibits the most linear and symmetric conductance update behavior.

**Fig. S16** Temperature-dependent optical switching dynamics and all-optical weight-update linearity. **a-c** Transient current relaxation curves measured at 298.15 K (a), 313.15 K (b), and 328.15 K (c), together with biexponential fitting results. **d-f** Normalized conductance evolution during optical potentiation under 980 nm illumination and optical depression under 525 nm illumination at 298.15 K (d), 313.15 K (e), and 328.15 K (f), respectively. The device maintains highly linear bidirectional weight updates over the tested temperature range.

**Fig. S17** Optical cycling endurance of the PbS/PEDOT:PSS/VOₓ device. Current evolution of the low- and high-conductance states during 1000 repeated optical switching cycles, showing stable state retention and a well-preserved current window.

**Fig. S18** Device-to-device uniformity of highly linear all-optical conductance update. **a** Optical potentiation and depression characteristics of five representative devices under identical optical pulse stimulation, showing highly linear and symmetric current modulation. **b** Statistical distribution of the modulation range extracted from 20 devices, indicating small device-to-device variation and good uniformity.

**Fig. S19** Highly linear multilevel current modulation with 8-bit resolution. Current evolution of the device under successive 980 nm optical pulses, showing 256 distinguishable states and nearly linear multilevel modulation.

**Fig. S20** Long-term retention stability of multilevel current states. Retention characteristics of selected programmed current states over 10^4^ s, showing stable and well-separated multilevel states.

**Fig. S21** Stability of 8-bit multilevel states under repeated optical programming cycles. Retention profiles of 256 optically programmed current states measured after the 1st, 10th, 20th, 30th, 40th, and 50th programming cycles, showing well-preserved state separation and stable multilevel current distributions.

**Fig. S22** Visualization of conductance maps and weight distributions of fully connected layers. **a** Conductance map of the first fully connected layer (FC1), where each pixel represents the mapped conductance value of a synaptic weight. **b** Weight distribution of FC1 before quantization. **c** Conductance map of the second fully connected layer (FC2), showing output-neuron-dependent conductance patterns. **d** Weight distribution of FC2 before quantization.

**Fig. S23** Optical images of the memristive crossbar array and PCB-level integration. **a** Photograph of the fabricated 32 × 32 memristive crossbar array mounted on a custom-designed printed circuit board (PCB), showing wire-bonded electrical connections between the array chip and peripheral pads. **b** Enlarged optical microscope image of a representative cross-point region, showing the well-defined intersection of the top and bottom electrode lines.

**Fig. S24** Schematic illustration of the optical programming geometry in the crossbar array. Localized light illumination is focused onto the cross-point region, where the thin top electrode and the exposed/electrode-edge active-layer regions allow optical interaction with the PbS/PEDOT:PSS/VOₓ heterostructure.

**Fig. S25** Hardware architecture of the DMD-assisted optical programming and electrically multiplexed readout platform. Schematic of the 32 × 32 memristor crossbar array integrated with DMD-based optical programming and FPGA-controlled electrical readout circuits.

**Fig. S26** Schematic illustration of the selected read path and possible sneak paths in the crossbar array. The selected cell is addressed through the target word line (WL) and bit line (BL), while unselected cells may contribute to parasitic leakage paths during readout.

**Fig. S27** Selected-cell and non-selected-cell current characterization in the 32 × 32 memristor array. **a** Comparison of the selected-cell current and the maximum non-selected-cell current under HRS, random 8-bit, and LRS background conditions. **b** Maximum leakage currents measured from half-selected and fully unselected cells under different array backgrounds. **c** Statistical distributions of the half-selected and fully unselected currents. The horizontal lines indicate the corresponding mean values. **d** Selected-to-maximum-non-selected current ratios under HRS, random 8-bit, and LRS background conditions.

**Fig. S28** Half-selected-cell leakage analysis in the 32 × 32 memristor array. **a** Current distribution along the selected row, where the target cell M16,16 is highlighted in red and the remaining bars represent half-selected cells sharing the same word line. **b** Current distribution along the selected column, where the target cell M16,16 is highlighted in red and the remaining bars represent half-selected cells sharing the same bit line. **c** Selected-to-maximum-half-selected current ratios for the same-row and same-column configurations. **d, e** Enlarged leakage-current distributions of the half-selected cells along the selected row (d) and selected column (e). **f** Average and maximum half-selected-cell leakage currents for the same-row and same-column configurations.

**Fig. S29** Read-voltage-dependent selected-cell and non-selected-cell current characteristics of the 32 × 32 memristor array. **a** Selected-cell current as a function of read voltage under HRS, random 8-bit, and LRS background conditions. **b** Maximum non-selected-cell current as a function of read voltage under the three array-background conditions. **c** Selected-to-maximum-non-selected current ratio as a function of read voltage. **d** Corresponding normalized read margin. The selected-cell signal remains clearly distinguishable from the maximum measured non-selected current over the tested read-voltage range of 1–100 mV.

**Fig. S30** Current-noise-floor characterization and minimum-resolvable conductance analysis. **a** Repeated-read current trace of a representative intermediate conductance state measured at a read voltage of 0.1 V, yielding an RMS current noise of approximately 5.3 nA. **b** RMS current-noise values extracted from representative HRS, mid-level, and LRS states. **c** Current distributions of two representative adjacent conductance states, Level 127 and Level 128. The horizontal lines indicate the corresponding mean current values. The adjacent-state current separation is 56.0 nA, exceeding the 3σ combined noise level of 24.3 nA. **d** Comparison between the minimum resolvable conductance difference determined using the 3σ criterion and the measured adjacent 8-bit conductance step.

**Fig. S31** Simplified 32 × 32 resistive-network SPICE simulation of distributed sneak-path contributions. **a** Schematic illustration of the 32 × 32 network model. The target cell is fixed at M16,16, with WL16 biased at 100 mV and BL16 connected to virtual ground. **b** Simulated equivalent distributed sneak currents for an LRS target cell under HRS, random 8-bit, and LRS background conditions. **c** Simulation parameter set used for the resistive-network model. **d** Selected-to-sneak current ratios for HRS and LRS target cells under different array-background conditions.

To provide a systematic benchmarking of the proposed device, Table S1 summarizes the key performance metrics of representative optoelectronic memory and synaptic devices reported in recent literature. Since different systems are often evaluated using different datasets, network architectures, and task settings, the comparison mainly focuses on operation modality, conductance linearity, energy consumption, array scale, and system-level hardware implementation, which are critical for practical neuromorphic vision applications.

**Table S1** Comparison of the optoelectronic memory devices based on different photoelectronic function materials

| Material  system | Fully  optical  operation | Retention | Conductance states | Linearity | Energy  consumption | Array scale | Full hardware system | Applications | Refs |
| --- | --- | --- | --- | --- | --- | --- | --- | --- | --- |
| ZnO/CsPbBr₃ | Yes | 10–100 s | Multilevel | - | - | 10×10 | No | Motion detection | [S1] |
| MSFP | No | ~50 s | 16 | - | - | 12×12 | Yes | Pattern recognition | [S2] |
| CuSCN/PM6:L8-BO/PFN-Br | Yes | >250 s | 120 | 0.53(AR) | 0.2 fJ | 26×26 | Yes | Predator evasion | [S3] |
| GTO/Al/HfO_x_ | No | - | 2^12^ | 0.992 | - | 4×4 | No | Logic operation | [S4] |
| BSO/LSMO/STO | No | ~10⁴ s | 8 | - | 1.36 fJ | 1×9 | Yes | RC digit recognition | [S5] |
| ZnO/OCNPs | Yes | - | >20 | 0.9993 | - | - | No | Motion detection | [S6] |
| Carbon/MoS₂ | No | ~5 s | 128 | - | 1.6 fJ | - | No | Edge detection | [S7] |
| DJ-V-HP | No | ~10^4^ s | 50 | 0.998 | 2.81 pJ | 7×7 | No | Image inference | [S8] |
| PbS/PEDOT:PSS/VOₓ | Yes | ~10^4^ s | 256 | 0.9994 | 0.63 fJ | 32×32 | Yes | BloodMNIST classification | This work |

**Supplementary References**

[S1] X. Han, J. Tao, Y. Liang, F. Guo, Z. Xu et al., Ultraweak light-modulated heterostructure with bidirectional photoresponse for static and dynamic image perception. Nat. Commun. **15**(1), 10430 (2024). <https://doi.org/10.1038/s41467-024-54845-3>
[S2] G. Zhou, J. Li, Q. Song, L. Wang, Z. Ren et al., Full hardware implementation of neuromorphic visual system based on multimodal optoelectronic resistive memory arrays for versatile image processing. Nat. Commun. **14**(1), 8489 (2023). <https://doi.org/10.1038/s41467-023-43944-2>
[S3] H. Lian, Z. Dou, Z. Qin, X. Cheng, Y. Ren et al., A polychromatic neuromorphic visual system inspired by biomimetics for miniature insect robots. Adv. Mater. **37**(35), e2416649 (2025). <https://doi.org/10.1002/adma.202416649>
[S4] P. Li, X. Shan, Y. Lin, Y. Du, J. Ma et al., Ultra-highly linear Ga₂O₃-based cascade heterojunctions optoelectronic synapse with thousands of conductance states for neuromorphic visual system. Light Sci. Appl. **14**(1), 354 (2025). <https://doi.org/10.1038/s41377-025-01897-9>
[S5] J. Xu, Y. Zhang, J. Wang, W. Zhang, T. Li et al., Ultra-low power and robust Bi₂SeO₅ films optoelectronic memristors for bio-visual perception computing systems. Adv. Mater. **37**(40), e09174 (2025). <https://doi.org/10.1002/adma.202509174>
[S6] J. Han, H. Mao, Y. Lin, K. Peng, L. Meng et al., Near-infrared optoelectronic memristor with all-optical modulation for microscopic biological motion recognition. Adv. Mater. **37**(45), e10185 (2025). <https://doi.org/10.1002/adma.202510185>
[S7] Y. Wang, L. Chen, B. Wu, J. Wang, T. Fu et al., Dual-mode optoelectronic neuromorphic memory for complex edge detection and recognition. Adv. Funct. Mater. **35**, 2514949 (2025). <https://doi.org/10.1002/adfm.202514949>
[S8] S.J. Kim, I.H. Im, J.H. Baek, S. Choi, S.H. Park et al., Linearly programmable two-dimensional halide perovskite memristor arrays for neuromorphic computing. Nat. Nanotechnol. **20**(1), 83–92 (2025). <https://doi.org/10.1038/s41565-024-01790-3>
[S9] W. Shockley, W.T. Read, Statistics of the recombinations of holes and electrons. Phys. Rev. 87(5), 835–842 (1952). https://doi.org/10.1103/PhysRev.87.835
[S10] R.N. Hall, Electron-hole recombination in germanium. Phys. Rev. **87**(2), 387 (1952). https://doi.org/10.1103/PhysRev.87.387
[S11] Y. Wang, E. Liu, A. Gao, T. Cao, M. Long et al., Negative photoconductance in van der Waals heterostructure-based floating gate phototransistor. ACS Nano **12**(9), 9513–9520 (2018). <https://doi.org/10.1021/acsnano.8b04885>
